# Third-generation cognitive behavioral therapy versus treatment-as-usual for attention deficit and hyperactivity disorder: a multicenter randomized controlled trial

**DOI:** 10.1186/s13063-021-05983-2

**Published:** 2022-01-28

**Authors:** Laetitia Crouzet, Anne Gramond, Carey Suehs, Pascale Fabbro-Peray, Mocrane Abbar, Jorge Lopez-Castroman

**Affiliations:** 1 Department of Psychiatry, Nimes University Hospital, Nimes, France; 2 Departments of Medical Information and Respiratory Diseases, Univ Montpellier, CHU Montpellier, Montpellier, France; 3 Department of Biostatistics, Epidemiology, Public Health and Medical Information, Nimes University Hospital, Nimes, France; 4 IGF, Univ. Montpellier, CNRS, INSERM, Montpellier, France; 5 CIBERSAM, Madrid, Spain

**Keywords:** ADHD, Psychosocial treatment, Barkley program, Randomized clinical trial

## Abstract

**Background:**

This study aims to compare improvements in attention deficit and hyperactivity disorder (ADHD) symptom severity between a group of ADHD children and parents undergoing a new therapeutic program based on third-generation cognitive behavioral therapy (Hyper-mCBT) and a similar group undergoing treatment-as-usual with the Barkley program.

**Methods:**

Two hundred forty-eight children diagnosed with ADHD will be randomly assigned to either a Hyper-mCBT program or a Barkley program. This is a multicenter randomized (1:1), 2 parallel-group, superiority trial with evaluator blinding and stratification according to center and methylphenidate treatment. The Hyper-mCBT program consists in a series of 16 simultaneous-but-separate therapy sessions for parents and for children.

**Discussion:**

More effective psychotherapeutic approaches are needed for ADHD children. Pharmacotherapy seems to be more effective in reducing ADHD symptoms but it is not always helpful, it carries side effects, and it is rejected by many parents/professionals. Results for psychotherapy programs for ADHD are inconsistent although several studies have shown clinical improvements. This trial will substantiate encouraging preliminary results of an innovative psychotherapy program for both parents and children.

**Trial registration:**

ClinicalTrials.gov NCT03437772. Registered on February 19, 2018. Sponsor number: PHRC-N/2016/JLC-01. RCB identification: 2017-A01349-44

**Supplementary Information:**

The online version contains supplementary material available at 10.1186/s13063-021-05983-2.

## Administrative information

Note: the numbers in curly brackets in this protocol refer to SPIRIT checklist item numbers. The order of the items has been modified to group similar items (see http://www.equator-network.org/reporting-guidelines/spirit-2013-statement-defining-standard-protocol-items-for-clinical-trials/).

**Table:** 

Title {1}	Third-generation cognitive behavioral therapy versus treatment-as-usual for attention deficit and hyperactivity disorder: a randomized, 2-parallel-group, evaluator blinded, superiority trial
Trial registration {2a and 2b}.	Clinicaltrials.gov registration: NCT03437772 (first posted: February 19, 2018). ClinicalTrials.Gov trial registry meets the WHO registry network criteria. WHO items are available at the registration link.
Protocol version {3}	Version 3.0 of this protocol was approved on 09/30/2019. Recruitment began on February 19th, 2018.
Funding {4}	Research grant by the French Government (“Programme Hospitalier de Recherche Clinique”)
Author details {5a}	Laetitia Crouzet, MSc. a, Anne Gramond, MD, a, Carey Suehs, PhD, b, Pascale Fabbro-Peray, MD, PhD, b, Mocrane Abbar, MD, PhD, a, Jorge Lopez-Castroman, MD, PhD, a,c,d. a Department of Psychiatry, Nimes University Hospital, Nimes, France b Department of biostatistics, epidemiology, public health and medical information, Nimes University Hospital, Nimes, France c IGF, Univ. Montpellier, CNRS, INSERM, Montpellier, France d CIBERSAM, Spain
Name and contact information for the trial sponsor {5b}	Nimes University Hospital, Department of Clinical Research, Partnerships and Medical Projects, email: drc@chu-nimes.fr
Role of sponsor {5c}	Funder and sponsor had no involvement in the design, organization, analysis or preparation for publication of the study, and no authority over any of these activities.

## Introduction

### Background and rationale {6a}

Attention deficit and hyperactivity disorder (ADHD) is a neurodevelopmental disorder characterized by abnormally elevated levels of inattention, impulsivity, and hyperactivity that cause functional impairment in the personal and socio-professional domains. According to a review gathering information from 103 studies across the globe, ADHD, as defined by the International Classification of Diseases (ICD-10), is present in about 5.3% of school-aged children [23]. Another review by the same group confirmed the stability of ADHD prevalence rates over the last 30 years [24]. It is interesting to note that ADHD represents 1/3 of consultations in child/adolescent psychiatric settings [29]. Apart from the behavioral and attentional problems that it causes, children and adolescents with ADHD have been qualified as socially disadvantaged, reporting low self-esteem and deficits in emotional and behavioral regulation [2]. In their adulthood, ADHD children present an increased risk of educational failure, unemployment, mental disorders, car accidents, substance abuse, interpersonal problems, and law-breaking behaviors [6, 7, 9, 18].

Concerning the ADHD treatment, international and French guidelines advocate a multidimensional approach. The psychosocial approach is used in the first intention. When ADHD symptomatology persists or worsens, medical treatment (methylphenidate) can be used in second intention. Most research has focused on drug treatments since the latter have shown higher efficacy in decreasing ADHD symptoms.

Treatment recommendations are based on a rich but very heterogeneous corpus of literature [11, 16, 22, 30]. The association of methylphenidate and cognitive behavioral therapy (CBT) seems to be superior to pharmacological treatment alone, favoring a reduction of ADHD symptoms and an improvement in global functioning [19, 21, 22, 25]. Indeed, the ADHD Observational Research in Europe (ADORE) study has shown that the most significant short-term effects were obtained using medications but over the long-term a combination with psychotherapy was more efficient [10]. However, there is a strong heterogeneity among studies, including different measures and raters and varying endpoints, that precludes the drawing of any firm conclusion [14] and stresses the importance of assessor-blinding for future studies [26].

In practice, the most widely used and studied psychosocial intervention is the parental training program [12]. Literature shows that such programs have a positive influence on parental well-being, parental ability to cope with child behavior problems, parent-child relationships, and child behaviors [1, 3, 4, 27].

Several reasons justify the interest of an integrated psychotherapy program that goes beyond parental guidance to improve ADHD symptoms in the long term. On the one hand, metanalytic reviews have only found partial support for nonpharmacological interventions for ADHD, either applied to parents or children, and they highlight the need for more research into psychological treatments [14, 28]. Previous studies have demonstrated the superiority of simultaneous care for both parents and children versus children or parents alone [15, 17]. On the other hand, the effectiveness of pharmacotherapy is often reduced, especially in the presence of comorbidities (80% of children with ADHD) and a growing number of parents/professionals are reluctant to try drug treatments. If they do try them, the effects of drug therapy diminish over time. Our preliminary data in a pilot non-controlled small sample (*n* = 30 unpublished) suggests that the use of last-generation CBT practices, such as mindfulness [20], in an integrated program for parents and children may be more effective than parental guidance alone. We have termed this program Hyper-mCBT, a unique combination of parental guidance with psychotherapeutic interventions for ADHD children and their parents.

### Objectives {7}

According to preliminary clinical data, we hypothesize that the Hyper-mCBT program will reduce the symptoms of ADHD and anxiety/depression scores, while improving the levels of self-esteem, emotional control, social integration, and academic achievement. The primary objective of this study is to compare improvements in ADHD symptom severity between a group of ADHD children and parents undergoing the Hyper-mCBT program and a similar group undergoing treatment-as-usual with the Barkley program. The evaluation of ADHD severity will be made at 5 and 8 months post-inclusion. The secondary objectives of this study are to compare the randomized groups concerning parenting styles and parental quality of life, anxiety and depression levels in both children and parents, and social well-being, school parameters, self-esteem, global functioning, and behavior in participating children.

### Trial design {8}

This is a randomized (1:1), two parallel-group, multicenter superiority trial with evaluator blinding and stratification according to center and methylphenidate treatment. Randomization is based on families, not on individual children.

## Methods: participants, interventions, and outcomes

### Study setting {9}

The trial takes place in several public psychiatric hospitals in France and is coordinated by the Nimes University Hospital. The clinical aspects of this trial take place within participating academic/public hospitals (urban setting) located in France: Nimes, Montpellier, and Paris.

### Eligibility criteria {10}

Participants are children between 7 and 15 years of age presenting an attention deficit and hyperactivity disorder (ADHD Rating Scale Parent Version: Investigator Administered and Scored ADHDRS-PI > 27). The patient and the parents (or legal guardians) give their informed consent and are insured or beneficiaries of a health insurance plan. Regarding treatment, the patient may either be treated with a stable dosage of methylphenidate (not expected to vary in the near future) and remain symptomatic or not be on treatment.

Participation in another trial or study that may interfere with the results or conclusions of this study is a criterion for exclusion, as well as being in a period of exclusion determined by a previous study. Participants whose parent (s) is (are) under judicial protection or is an adult under guardianship are also excluded from the study. In addition, the family cannot be included if it is impossible to properly inform the patient, his/her parents, or legal guardian, and if the patient or parents refuse participation, signature of signed consent, or follow-up procedures. If the investigator suspects the presence of or if there is documented information about an intellectual disability (*IQ* < 70), the patient is excluded. The same applies to patients diagnosed with autism spectrum disorder, psychotic disorder, or bipolar disorder. Finally, the patient should not be involved in cognitive behavioral therapy (individual or group) during the 6 months prior to inclusion and/or have previously participated in this study. Written informed consent is obtained from all participants by the investigators (senior psychiatrists or psychologists) after explaining the purpose and methodology of the study (please see “Declarations”/“Ethics” for more details).

### Who will take informed consent? {26a}

Participation in the study is systematically proposed to all ADHD children/adolescents and their parents meeting eligibility criteria. The study is presented to patients and their parents as a clinical trial to compare treatments with similar benefits, along with appropriate information letters. Oral and written information about the study is adapted to the age of the patients (6–10 and 11–15 years). Trained psychiatrists and psychologists participating in the study are responsible for obtaining consent. If the patients/parents show interest in the study, the first evaluation visit is organized. Please see Ethics approval and consent to participate {24} for further details.

### Additional consent provisions for the collection and use of participant data and biological specimens {26b}

Not applicable. Participant data will not be used in any ancillary studies. This trial does not involve collecting biological specimens for storage.

## Interventions

### Explanation for the choice of comparators {6b}

Parental guidance programs are part of French [11] and international [16, 30] recommendations for the care of ADHD children. We selected the Barkley program as a comparator since it is the best-known and studied parental training program [5] and there is consistent evidence on its benefits [13].

### Intervention description {11a}

Interventions take place during the school year to avoid perceived seasonal differences in children’s stress levels (among others). At the end of the first evaluation visit, randomization occurs to assign the patient either to the experimental group (mCBT) or the comparator group (treatment as usual (TAU): Barkley therapy). At this time, only a designated person in charge of organizing therapy sessions is aware of the randomization results.

#### Experimental group (mCBT)

The mCBT program is a 3rd-generation cognitive behavioral therapy program that combines social skills training, emotional regulation, self-esteem, cognitive remediation, and mindfulness therapies for children and behavioral techniques, emotional regulation, and mindfulness for parents. This program consists in a series of 16 simultaneous-but-separate therapy sessions for parents and for children (see Supplementary Table 1 for details). Each of the 2 × 16 sessions will last 75 min every week. To facilitate the participation of children, the duration of the sessions was reduced to 75 min, but the total amount of therapy during the program is similar to the comparator group. Parents participate in the parental guidance program with their therapist (psychologist or psychiatrist), and children participate in their own program with their own therapists (1 leader and 1–2 regulators). The simultaneous nature of the program avoids excessive impact on the daily life of families. The therapists are trained in cognitive behavioral therapy.

##### Children group

The first therapist or “leader” runs the session and is considered as the teacher or “facilitator”; the second therapist, the “regulator,” manages the behavior of the children and the group and the organizational aspects. When many children are present, it may be appropriate for three adults to be present, with a second adult regulating “disruptive” behavior and accompanying some children back to calmness while the first regulator concentrates on reinforcement activities.

Children’s groups are formed according to age and school level in order to encourage a sentiment of “belonging.” The vocabulary and techniques used are adapted to each group’s age level and cognitive abilities. The place where children’s sessions take place is important. The room must be simple with few distractors. Seating should be arranged in a U shape to facilitate interaction. Seating should also be quiet in nature (e.g., put tennis balls on chair legs to avoid scraping noises). The session leader is positioned in front of the children next to a paperboard or a slideshow. The regulator freely moves around the room as required or preferentially stays near the back. Seating arrangements for children should be discussed before sessions in order to avoid unwanted interactions. During such sessions with ADHD children, the participating therapists must allow for a certain amount of acceptable agitation and define strategies that help children self-regulate their behavior.

The therapists are encouraged to use a timer. The first 60 min is dedicated to the session, and the last 15 to a summary and review of material covered during the session in order to help emphasize the overall take-home message, to the children’s mission to be performed at home or school, and to self-evaluation and finally an end-of-session game. A bell can be used to signal the beginning and end of each activity, mediation time, etc.

All sessions present the same structure: the group starts with a reminder about the rules, after the mediation “today’s weather and stress thermometer,” then a review of the previous session’s missions. Then comes the sequence of the day which must have a visual support (slides or posters) describing the content of the session. The session ends with a session summary, the new missions, and the child makes a self-evaluation of his behavior. To conclude, there is an end-of-session game.

##### Parent session

This program is largely inspired by the Russel Barkley program into which have been integrated other tools like mindfulness, acceptance and commitment therapies (ACT), and nonviolent resistance. We adapted these therapeutic techniques in our clinical practice and recommend that they be coupled with a support group for children with ADHD.

The aim of the integrative therapeutic parent group is to offer 16 sessions of integrative therapy on the various spheres of life of the child affected by ADD/ADHD to promote a better quality of life and well-being for the child and family. The parent is placed in a co-therapist position, pledging to initiate change in his/her relationship with his/her child. He/she should not expect an immediate positive reaction from the child. The parent has the responsibility of avoiding any manifestation of verbal or physical violence. Sessions with parents are led by a single therapist trained in cognitive behavioral therapy. Therapists are encouraged to use a visual time cue, such as a timer. The timer can give an indication of the time remaining in the session. For a 75-min session, the first 30 min is devoted to a review of the tasks performed and the last 45 min is devoted to the meeting and presentation of new missions. The place where parental sessions take place is important. The layout of the environment is essential to ensure optimal attentional mobilization, to promote exchanges not only between therapist and parents but also among parents. The room is uncluttered with few distractors, the seats are installed in a U to promote interaction. The therapist is positioned in front of the parents before the paperboard or Powerpoint. The structure of a typical session is described in Table 1.

**Table 1:** Structure of a typical session of the Hyper-mCBT program for parents

Structure of the session	
1	Welcome
2	Meditation: today’s interior weather
3	Review of last session’s missions
4	Relaxation
5	Session: the day’s theme
6	Session summary
7	New missions
8	Summary and missions presented in children’s group

#### Control group (Barkley program)

The Barkley program is specifically conceived for parents of children with ADHD, either individually or in groups of 6 to 8 families, with twelve 90-min sessions, occurring twice a month. The objective of this program is to train parents to cope with the difficult situations they encounter, to learn effective control strategies that are coherent and adapted to the “deviant” behavior of their children in order to reduce the intensity of events and their consequences on family life. All this inevitably involves the improvement of family relationships through an essential improvement of the image that parents have of themselves, the image the child has of him/herself, and overall family functioning.

Each session focuses on a “theme” or a particular situation: practical exercises are offered to families to improve communication, adjust the educational responses, analyze the behavior of the child, and anticipate crises in order to avoid them. These practical exercises are practiced at home. A typical session has the following structure: a 30-min review of exercises practiced at home, 60 min: theme of the day’s and new exercises.

The control group facilitator cannot participate in mCBT therapy to avoid bias. The Barkley program is already in use by all participating centers and training per se is not required. Nevertheless, a standardized consensus has been established between the centers to reduce variability before beginning the study and regular meetings are made annually to ensure that the program is followed faithfully.

### Criteria for discontinuing or modifying allocated interventions {11b}

Not applicable. There will be no special criteria for discontinuing or modifying allocated interventions.

### Strategies to improve adherence to interventions {11c}

Participation in each group session is registered using a sign-up sheet in order to compare adherence between the groups. We use only one strategy to improve adherence, which consists in a phone call to the parents in case of repeated (more than 2) nonappearances. The same method is used in both arms.

### Relevant concomitant care permitted or prohibited during the trial {11d}

Regarding treatment, the patient may either be treated with a stable dosage of methylphenidate (not expected to vary in the near future) and remain symptomatic or not be on treatment. The patient should not be involved in cognitive behavioral therapy (individual or group) during the 6 months prior to inclusion.

### Provisions for post-trial care {30}

The subjects included in the trial are monitored for 8 months. The monitoring of complications and adverse events is scheduled. Any patients who experience an adverse event (or not) are followed up by the investigator until complete resolution of the complication. Following study completion or end, follow-up is continued as decided by the investigator.

### Outcomes {12}

Three evaluation times are realized (inclusion, after 5 months, and after 8 months). During the evaluation carried out by a blinded evaluator, questionnaires and self-questionnaires are carried out:

The first objective of this research is evaluated by the ADHDRS-PI questionnaire in an interview with the parent(s) and the patient. The 18 items correspond to the 18 symptoms listed in the DSM-IV ADHD diagnosis. The reliability and validity of the ADHDRS-PI have been studied in a panel of European countries, including France (Zhang et al. 2005; Döpfner et al. 2006).

Several secondary outcomes are evaluated:

- A: *The parental authority questionnaire* (PAQ) auto-questionnaire to compare changes in parenting styles between groups. It consists of 30 items per parent and yields permissive, authoritarian, and authoritative scores for both the mother and father (Buri 1991).

- B: *Parental–Developmental Disorders–Quality of Life* (PAR-DD-Qol). To compare changes in the quality of life for parents between groups. This auto-questionnaire contains 17 items (Raysse 2011).

- C: To compare changes in global function between groups:

*The Children’s Global Assessment Scale* (CGAS): The CGAS was designed by Shaffer et al. (1983) based on the Global Assessment Scale (Endicott et al. 1976) and was subsequently found to have discriminant and concurrent validity (Bird et al. 1987). It is widely used today as a clinician-rated scale assessing the overall functioning of a child based on all available information (Lundh et al. 2010).

*The Clinical Global Impressions Scale* (CGI-S): Is a 7-point scale used by an investigator to rank the severity of illness observed for a given patient. The CGI-S was developed for use in clinical trials to provide a brief, stand-alone assessment of the clinician’s view of the patient’s global functioning prior to and after initiating a study treatment.

- D: To compare changes in social well-being and school parameters for children between groups: *CONNERS 3*^*rd*^ *Edition for school teachers*. The evaluation of the child’s behavior is performed using the Conners questionnaire for teachers (Conners 2008).

- E: To compare changes in anxiety and depression (for both children and parents) between groups. *Multidimensional anxiety scale for children in 10 items* (MASC-10): The MASC items approximate the DSM-IV anxiety diagnoses and contain four factors: physical symptoms (tense/somatic), a harm avoidance (perfectionism/anxious coping), a social anxiety (humiliation/performance fears), and a separation anxiety/panic. The full MASC has shown good internal consistency and test-retest reliability (March et al. 1997, 1999; March & Sullivan 1999; Rynn et al. 2006; Baldwin & Dadds 2007).

*Children depression inventory 2* (CDI2-short version): An evaluation of a depressive episode is performed using the Children’s Depression Inventory, a hetero-questionnaire developed by Bae (2012). This is a 12-item questionnaire which assesses depression in children of 7 to 17 years of age.

*Hospital anxiety and depression scale* (HADS): An auto-questionnaire for adults, developed by Zigmond & Snaith (1983), Snaith & Zigmond (1986), Herrmann (1997), Bjelland et al*.* (2002), and Snaith (2003), which is validated in French (Lépine et al. 1985; Untas et al. 2009), is commonly used to screen for anxiety and depressive disorders in clinical studies. The HADS questionnaire has 14 questions, including 7 questions concerning anxiety and 7 concerning depression.

- F: To compare changes in self-esteem and behavior for children.

*Rosenberg Scale*: The auto-questionnaire is for children, which measures global self-esteem and is composed of 10 items for which the subject must give his/her level of agreement (Rosenberg 1965).

*CONNERS 3*^*rd*^ *Edition for parents*: The evaluation of the child’s behavior is performed using the Conners auto-questionnaire for parents. This is a behavioral observation questionnaire for children from 6 to 18 years of age (Keith Conners 2008).

And to finish, two diagnostic questionnaires are used:

*The Schedule for Affective Disorders and Schizophrenia for School-Age Children – Present and Lifetime Version* (K-SADS-PL). This is a semi-structured diagnostic interview that assesses current and past episodes of psychopathology in children and adolescents according to DSM-III-R and DSM-IV criteria. Published study results indicate that the K-SADS-PL generates reliable and valid child psychiatric diagnoses (Kaufman et al. 1997).

*Adult ADHD self-report scale* (ASRS) was designed by researchers from the New York University and Harvard medical schools in collaboration with the World Health Organization (WHO). The ASRS is an auto-questionnaire composed of eighteen questions which reflect DSM-IV criteria and address current ADHD symptoms in adults (Kessler et al. 2005; Adler et al. 2006).

### Participant timeline {13}

**Fig. 1:**
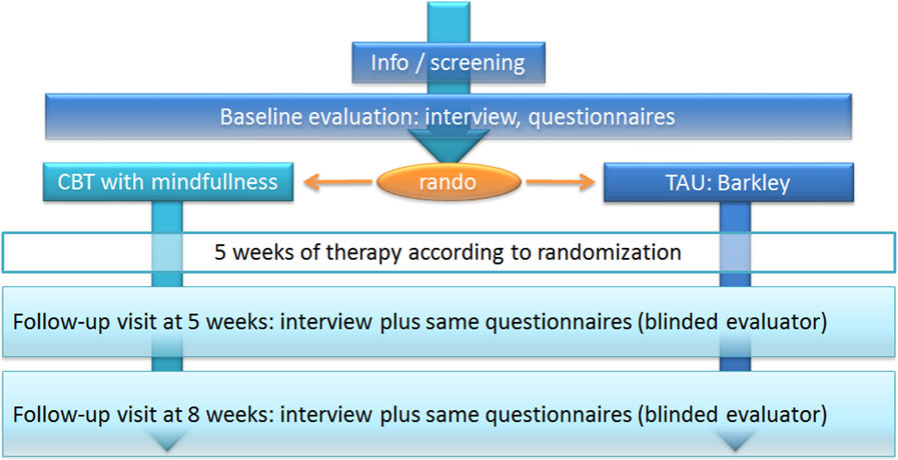
Study design

**Fig. 2:**
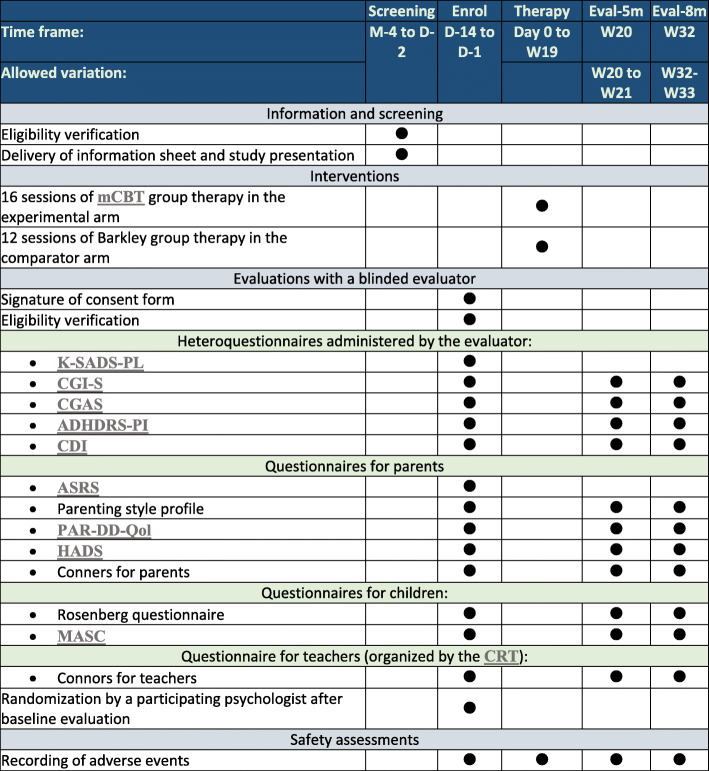
Participant timeline

The study is presented to children and their parents during a selection visit, along with appropriate information letters. If the children/parents show interest in the study, the 1st evaluation visit for the parents and children is organized and their group assignment occurs. Therapy begins after randomization and lasts about 5 months. Then evaluation visits at 5 months (after therapy) and 8 months (post-therapy evolution) are planned. The timeline of the therapy is summarized in Figs. 1 and 2.

### Sample size {14}

We could not find prior studies on psychological interventions for ADHD using a similar sample, assessments, and research outcomes. Thus, to estimate the sample size, we used the following: (i) preliminary data collected from a pilot study on the experimental intervention (*n* = 30, personal data) and (ii) previous data on the efficacy of the Barkley program on a large sample (van den Hoofdakker et al., 2007). We compared the change in the number of patients fulfilling the diagnostic criteria for ADHD in both samples from baseline to the end of the therapy, although the assessment instruments were different (ADHD Rating Scale in our sample versus the Conners Parents Rating Scale).

To highlight a difference of relative reduction of risk of 9% (*SD* = 20.6%) between the two groups: 15% parental guidance (van den Hoofdakker et al. 2007) versus 24% mCBT (Gramond, personal data), with a two-sided alpha risk of 5% and a power of 90%, it is necessary to recruit 111 children per group. To take account of a potential 10% loss during follow-up, 124 children per group will be recruited, i.e., 248 children. The statistical unit corresponds to a family, which includes a calculated sample size of children with ADHD plus one or both parents.

### Recruitment {15}

Approximately 650 families per year consult at Nimes University Hospital requesting help in dealing with an ADHD child. As this is the smallest establishment participating in this protocol, we expect larger potential recruitment pools for the other centers. Our target rhythm for inclusions is 1–2 families per month and per center (on average).

This is largely inferior to potential recruitment pools in order to take into account the time constraints associated with randomized group therapy, the need to implement the interventions during the school year, and the possibility that certain families may choose not to participate and also to have a feasible inclusion curve.

## Assignment of interventions: allocation

### Sequence generation {16a}

Families are randomized to either study arm in a 1:1 ratio. Randomization lists consisting of centralized randomly sized blocks are established per center. These lists are the responsibility of an independent methodologist at the BESPIM. A specifically designed SAS program (Cary, NC, USA) is used to carry out randomization.

### Concealment mechanism {16b}

Patient inclusions are performed via an online software called “Inclusio” (inclusio.bespim.fr), an inclusion-randomization software designed for clinical research projects. Following user login, patient identification (first letter of last name + first letter of first name + year of birth) and verification of screening and exclusion criteria, the treatment number for blind studies, or the study arm for open-label studies are indicated to the user. It is impossible to modify the order of randomization; patient assignment to a study arm and a randomization number is definitive. The program provides real-time inclusion alerts to study staff requesting such alerts.

### Implementation {16c}

The allocation sequence is generated by an independent methodologist at the BESPIM. Patient enrolment is carried out by including psychiatrists. Randomization is carried out after patient inclusion and after baseline assessments by participating psychologists (i.e., not the including psychiatrists, who are also the outcome assessors). The e-santé team at the BESPIM is in charge of setting up Inclusio for the needs of the project (note: statistical analyses are carried out at the family level and safety reporting is carried out at the individual level).

## Assignment of interventions: blinding

### Who will be blinded {17a}

Baseline assessments are made before randomization, so these are blinded.

Due to the nature of the intervention, neither participants nor staff can be blinded to allocation, but are strongly inculcated not to disclose the allocation status of the participant at the follow-up assessments. Patients cannot be blinded, but are asked to not reveal their group status to anybody outside their group, not even their treating psychiatrist. Furthermore, the hypotheses tested are not communicated to patients (i.e., the patients are not informed on the supposed superiority of one group over another). Therapy care providers (psychologists) cannot be blinded. In order to make assessments as objective as possible, outcome assessors (psychiatrists) are different from the therapy providers (psychologists), and every attempt is made to keep outcome assessors blinded to patient group status. To control for the success of blinding, a “guess-the-group” question is addressed to outcome assessors. Outcome assessor responses are compared to expected results due to chance (see the Statistical methods section).

Data analysts will not be involved in trial field logistics and will be blinded. During analyses, when group assignments are first required they will only be revealed as “group A” or “group B”. Only when analyses have been completed will the exact nature of groups be revealed.

### Procedure for unblinding if needed {17b}

Not applicable, the design is open label with only outcome assessors being blinded so unblinding will not occur.

## Data collection and management

### Plans for assessment and collection of outcomes {18a}

Clinical observations are recorded in the case report form as the study progresses.

The instruments/questionnaires to be administered:

1. Schedule for Affective Disorders and Schizophrenia for School-Age Children–Present and Lifetime Version (K-SADS-PL)

2. The Clinical Global Impression Scale (CGI-S)

3. The Children’s Global Assessment Scale (CGAS)

4. ADHDRS-PI

5. Children’s Depression Inventory (Kovacs 1981)

6. Adult ADHD Self-Report Scale (ASRS)

7. the Parental Authority Questionnaire (PAQ)

8. Parental–Developmental Disorders–Quality of Life (PAR-DD-Qol)

9. Hospital Anxiety and Depression Scale (HADS)

10. The Conners questionnaire for parents

11. Multidimensional Anxiety Scale for Children (MASC)

12. Rosenberg Self-Esteem Scale

13. The Conners questionnaire for teachers

Please refer to the “Outcomes {12}” section for a description of the instruments and references.

### Plans to promote participant retention and complete follow-up {18b}

The study clinical research technician maintains contact with families and teachers. Study calendars are distributed to families as early as possible in the study and kept up-to-date by the study clinical research technician. Reminders are sent to families in the week preceding each evaluation.

### Data management {19}

Performed in line with the international conference on harmonization of technical requirements for registration of pharmaceuticals for human use (ICH). The related documents are stored on the Department of Biostatistics, Epidemiology, Public Health and Medical Information at the Nimes University Hospital (BESPIM). Electronic case report form (eCRF) fields are formatted so as to enforce homogenous value types and require confirmation especially for out-of-expected-range values. All modifications are fully traceable (who, when, why?) to allow a complete audit trail. An electronic signature by the investigator engages his/her responsibility. The software used to create eCRFs is hosted on a website within Nimes University Hospital. Access to this software is secured via a password. The data collected through generated eCRFs are subject to daily backup on a secure network. The network is connected to the Internet; access is protected by a firewall. Clinical study data is stored in a specific directory on the server. Only network administrators and BESPIM authorized professionals have access to this directory.

Data management and statistical analysis are provided by the BESPIM. The conditions of transfer of all or part of the research database are decided by the research sponsor and are subject to a written contract.

The following measures are taken to implement confidentiality:

- The required information technology is located at the BESPIM; access is controlled and secured.
- Data are stored on a server hosted in a secure room at Nimes University Hospital.

In case of hardware or software problems, a specific safety procedure has been implemented.

The export of data for analysis is conducted by a BESPIM authorized professional.

The closing of the trial including the closure of the centers is conducted in accordance with Good Clinical Practice and ICH. Medical and administrative records and CRFs are kept for the duration of the study in the service and then archived for a minimum of 30 years.

### Confidentiality {27}

In accordance with article R.5120 of the French Public Health Code, the investigators, as well as any persons collaborating in the study, will respect medical confidentiality especially as concerns the nature of the study, the persons participating in the study, and the obtained results.

The study protocol, documentation, data, and all other information generated are held in strict confidence. No information concerning the study or the data will be released to any unauthorized third party without prior written approval of the sponsor.

The investigator will ensure that the anonymity of each person involved in the study is respected. On all study-related documents, the patient is identified using only a unique, 7-character identification number, and the first letter of his/her last name, the first letter of his/her first name, and his/her year of birth. A patient identification list is maintained by the investigator (and only the investigator).

### Plans for collection, laboratory evaluation, and storage of biological specimens for genetic or molecular analysis in this trial/future use {33}

Not applicable. There will be no biologic specimens collected for storage.

## Statistical methods

### Statistical methods for primary and secondary outcomes {20a}

The primary outcome is the change from baseline in the ADHDRS-PI at 5 months. If required for meeting normality, the ADHDRS-PI scores will be appropriately transformed (e.g., Box-Cox transformation). A Student’s test will be used to compare the two groups. If the conditions for use of a Student’s test are not met, a non-parametric Mann-Whitney test will be used. This analysis will be completed by a modeling analysis to take into account clustering effects. Indeed, in our study, randomization to treatment is done on an individual basis; however, the experimental and control treatments are administered to a group so that several individuals receive the intervention together by the same therapists; the observations within the group therapy will likely be correlated within groups (clustering effect). We will use multilevel mixed-effects models to assess the treatment effect on the primary outcome: the ADHDRS-PI score at 5 months by adjusting for cluster effects and ADHDRS-PI score at baseline.

There is no a priori reason for carrying out per-protocol analyses in the present study. All analyses will therefore be performed on the intention-to-treat population. The level of significance is set at *p* < 0.05 (bilateral). The statistical analysis will be performed by the BESPIM using SAS software (SAS Institute, Cary, NC, USA) version 9.4 (or higher) or the R statistics environment (R Development Core Team 2008) version 3.3.1 (or higher).

### Interim analyses {21b}

Not applicable; no interim analyses are planned to avoid the possibility of a type 1 statistical error.

### Methods for additional analyses (e.g., subgroup analyses) {20b}

The models will also provide valuable estimates of intra-cluster correlation coefficients for the different outcomes of our study in the context of behavioral group therapy; these data are necessary to optimize the sample size of further studies in the area of psychological research. Similar methods will be used for secondary outcomes. The temporal evolution of repeated quantitative measures (baseline, 5 months, 8 months) will be compared between groups by a mixed model for repeated longitudinal data.

### Methods in analysis to handle protocol non-adherence and any statistical methods to handle missing data {20c}

Any deviations, reasons for such deviations, and all alternative or additional statistical analyses that may be done will be described in the final report.

As concerns the primary efficacy outcome, data missingness will probably not be random (MNAR). Multiple imputation methods will be used to replace missing data. For exploratory variables, missing data will not be replaced. Blinding will be removed in 2 steps: analysis will be performed upon completion of the study and freezing of the database, using patient group assignments as group A versus B only. When all analyses have been performed and the final report drafted, treatment assignment to groups will be fully unblinded. To control for the success of blinding, outcome assessor responses to the “guess-the-group” question will be compared to true responses using the kappa agreement coefficient.

### Plans to give access to the full protocol, participant-level data, and statistical code {31c}

Data management and statistical analysis is provided by the “Laboratoire de Biostatistique, Epidémiologie clinique, Santé Publique Innovation et Méthodologie” (BESPIM) at NUH. The conditions of transfer of all or part of the research database are decided by the research sponsor and are subject to a written contract. The data will not be publicly available.

## Oversight and monitoring

### Composition of the coordinating center and trial steering committee {5d}

The coordinating center is composed of the principal investigator and associated clinicians of the Child and Adolescent Psychiatry Department, as well as the research team (responsible for monitoring, data management, and statistical analysis) of the Department of Biostatistics, Epidemiology, Public Health and Medical Information (BESPIM) at the Nimes University Hospital. The trial steering committee is composed of the principal investigator (JLC), the investigator responsible for data management and analysis (PFP), and a study coordinator (Leonie Gazel).

### Composition of the data monitoring committee, its role, and reporting structure {21a}

Due to the low level of risk added by this research and the lack of interim analyses, a data monitoring committee (DMC) will not be formed.

### Adverse event reporting and harms {22}

The present study compares two types of group therapy for parents and children. Harms are not expected in association with group therapy. All the children and parents participating in group therapy are expected to positively benefit from said therapy. No specific surveillance is required. The only procedure added for research purposes is the administration of questionnaires.

Consequently, any serious adverse event occurring in a subject included in this research must be notified by the investigator to the appropriate safety system:

✓ Some children included in this protocol may be treated with Ritalin. In this case, any adverse effect likely to be due to the treatment administered or to its use must be declared to the Regional Center of Pharmacovigilance (CRPV)

✓ Any serious adverse event associated with the care must be reported by the investigator as part of the reporting obligation for serious adverse events related to care according to the procedures in effect in the institution

When the event is reported to the appropriate safety system, the investigator must specify the inclusion of the patient in a clinical research protocol, specifying the references of the research.

### Frequency and plans for auditing trial conduct {23}

A sponsor-delegated research assistant regularly visits each of the study centers during the implementation of the trial at least once a year. One or more visits are carried out during the trial according to the rhythm of the inclusions and the duration of the study. All monitoring visits are accompanied by a written monitoring report (visit traceability).

Investigators agree to comply with the requirements of the sponsor and the Competent Authority in respect to audits or inspections of the study. An audit can cover all stages of the study, from protocol development to publication of results and the classification of the data used or produced as part of the study

### Plans for communicating important protocol amendments to relevant parties (e.g., trial participants, ethical committees) {25}

Any substantial change, that is to say, any changes that might have a significant impact on the protection of persons, the conditions of validity and the results of research, on the quality and safety of the interventions tested, on interpretation of scientific documents that support the conduct of research, or the modality of conduct, will be the subject of a written amendment that is submitted by the sponsor to the Committee for the Protection of Persons (CPP) and the competent authority for approval prior to being implemented. Insubstantial changes, that is to say, those that have no significant impact on any aspect of research whatsoever, are transmitted to the CPP in order to inform the CPP of such changes.

All amendments to the protocol must be brought to the attention of all investigators involved in the research. Investigators are obliged to respect their content. Any amendment that modifies the care of patients or the benefits, harms, risks, and constraints of the research is the subject of a new briefing note and a new consent form which requires the same collecting procedures as mentioned above.

## Dissemination plans {31a}

Communication of results to participants, healthcare professionals, and the public will be made through scientific conferences and publications in “open-access.” Pursuant to Act No. 2002-303 of 4 March 2002, patients will be informed, upon request, of overall research results. Any written or oral communication of research results will receive prior approval from the coordinating investigator. Currently, Nimes University Hospital does not support public access to trial documents. However, should such requirements occur in association with publication submissions, the Open Science Framework will be used (https://osf.io/). All persons qualifying as authors according to the ICMJE will be asked to sign an authorship contract.

## Discussion

This project is designed to test an enhanced psychotherapy for children/adolescents with ADHD. Despite the high prevalence of this disorder in childhood, most research has focused on drug treatments since the latter have shown higher efficacy in decreasing ADHD symptoms. However, several reasons justify our project: (1) drugs are associated with short- and (unknown) long-term risks and adverse effects; (2) combined therapies (psychotherapy+stimulants) have better outcomes than stimulants alone; (3) either effect (drug or psychotherapy) diminishes with time, so new therapeutic approaches with long-term effects are therefore needed; (4) an increasing number of parents/professionals are reluctant to try drug treatments and require alternative approaches; and (5) the efficacy of pharmacotherapy is reduced in many cases, especially when comorbidities are present (80% of ADHD children).

To date, results for psychotherapy programs for ADHD are inconsistent although several studies have shown clinical improvements [14, 26]. This is probably due to heterogeneous measures and raters, small samples, and varying endpoints selected in prior studies. Clarifying the efficacy of specific psychotherapies for ADHD and standardizing their practice is therefore urgently needed. In the present day, care options for ADHD children vary and are strongly influenced by psychodynamic approaches in France.

During the elaboration of the mCBT program, we have observed reductions in ADHD symptomatology and anxiety-depression scores, but also an improvement of self-esteem, emotional regulation, social integration, and school results after mCBT therapy. This project will therefore implement a rigorous methodology in order to confirm preliminary results. This is an original program that integrates for the first-time multiple treatment approaches (social skills training, emotional and behavioral regulation, self-esteem, cognitive remediation, and mindfulness) into a single cognitive behavioral therapy (and not separate therapies). The program also integrates mindfulness, known to reduce ADHD symptoms and behavioral problems in children when tested independently of other interventions [8]. Finally, the program is carried out simultaneously for children and parents (including behavioral techniques, emotional regulation, and mindfulness as well), thus avoiding excessive impact on the daily life of families.

ADHD is a chronic pathology with major public health implications. Approximately 1/3 of ADHD children will not finish secondary studies, and many of them will show negative outcomes in their adult life, from unemployment or substance abuse to antisocial personality, marginalization, and criminality. The validation of a high-performing psychotherapeutic program, as we propose in this study, for minimizing the burden (huge personal, social, and economic costs) associated with ADHD is long awaited for.

## Trial status

Currently recruiting, 171 children/families have agreed to participate in three centers: Nimes, Montpelier, and Paris. Version 3.0 of this protocol was approved on 09/30/2019. Recruitment began on February 19, 2018. The expected date to complete the recruitment is March 2022.

## Supplementary Information

**Additional file 1: Supplementary Table 1.** Overview mCBT sessions for parents and children.

## Abbreviations

ACT: Acceptance and commitment therapies
ADHD: Attention deficit and hyperactivity disorder
ADHDRS-PI: ADHD Rating Scale Parent Version: Investigator Administered and Scored
CBT: Cognitive behavioral therapy
CPP: Committee for the Protection of Persons
DMC: Data Monitoring Committee
BESPIM: Department of Biostatistics, Epidemiology, Public Health and Medical Information at the Nimes University Hospital
DSM-IV: Diagnostic and Statistical Manual of Mental Disorders
eCRF: Electronic case report form
ICD-10: International Classification of Diseases
CRPV: Regional Center of Pharmacovigilance
TAU: Treatment as usual
