# Supplementary material for: Third-generation cognitive behavioral therapy versus treatment-as-usual for attention deficit and hyperactivity disorder: a multicenter randomized controlled trial
Source: Trials. 2022 Jan 28;23:83. doi: 10.1186/s13063-021-05983-2 (PMC8796622; doi:10.1186/s13063-021-05983-2)
Supplement: Supplementary file 1 — Additional file 1: Supplementary Table 1. Overview mCBT sessions for parents and children. [file 13063_2021_5983_MOESM1_ESM.docx]

Supplementary table 1. Overview of mCBT sessions for parents and children

| Session | Parents | Children |
| --- | --- | --- |
| 1 | **Group and program presentation**  Objective: Presentation of parents and their children, program objectives and organization. | **Group and program presentation**  Objective: Program presentation and objectives. How the group will function. Teach children how to actively present themselves to others with appropriate posture and involvement. Children learn to approach others without fear and to improve their social skills. |
| 2 | **ADHD psychoeducation**  Objective: This is a theoretical-knowledge-based session whose goal is to teach parents about their child’s disorder and how it affects different aspects of their child’s life (family, social situations, schooling). | **ADHD psychoeducation**  Objective: Transmission of knowledge concerning ADHD and associated comorbidities (anxiety and relational, emotional or organization difficulties). |
| 3 | **ADHD psychoeducation + comorbidities**  Objective: This is a continuation of presentation of information concerning ADHD, plus the most frequently associated comorbidities. | **Emotion definitions**  Objective: This session is dedicated to defining various emotions and recognizing the corresponding facial expressions. |
| 4 | **Psychoeducation about stress and initiation to Mindfulness**  Objective: Understand stress and how it affects the ADHD child and his/her family. Initiation to Mindfulness. | **The feeling and expression of emotions**  Objective: This session is dedicated to recognizing expressions and emotions, as well as the corresponding physical symptoms. |
| 5 | **The different styles of parenting**  Objective: To know the different parenting styles and how they affect children. Teach parents how to develop their democratic style. | **Stress management**  Objective: Psychoeducation about stress and relaxation-meditation techniques |
| 6 | **Improving relationships with your child**  Objective: To improve intra-family relationships, to learn how to make efficient requests and how to spend quality time with one’s child. | **Anger management**  Objective: Learning how to deal with anger. |
| 7 | **Improving the self-esteem of your child**  Objective: Provide parents with information on the development of self-esteem in children and adolescents. Provide therapeutic tools that favour good self-esteem in ADHD children/adolescents. | **Self esteem**  Objective: Recognizing worth in oneself and others. |
| 8 | **Oppositional behaviours in ADHD children/adolescents**  Objective: To understand opposition / provocation behaviours in ADHD children/adolescents are a bi-directional process that involves parents as much as children. The way in which a parent acts influences the behaviour of their child. | **Asserting oneself and anxiety**  Objective: understanding anxious and assertive behaviours. |
| 9 | **Resolving problems and conflicts**  Objective: Learn how to formulate effective criticism, manage conflicts and techniques for resolving problems. | **Giving and receiving compliments**  Objective: Learn how to give and receive compliments. |
| 10 | **Improving time management, poor sleeping habits and rhythms associated with ADHD**  Objective: To improve how the ADHD child perceives time management. Trouble sleeping is addressed in the second part of the session. | **Techniques for resolving problems and conflicts (social and cognitive)**  Objective: Managing conflicts at home (parents, brothers and sisters) and at school (with colleagues). |
| 11 | **Motivation tables**  Objective: Establish techniques aimed at positively reinforcing good behaviour in ADHD children. | **Responding to insults**  Objective: Learn how to appropriately respond to insults. |
| 12 | **Learning how to manage my child’s behavioural problems**  Objective: Teach parents how to manage inappropriate behaviour in an ADHD child. | **Facing harassment**  Objective: This session is dedicated to harassment and cyber-harassment. |
| 13 | **Managing outside behavioural problems**  Objective: To learn to manage outside behavioural problems and to be less sensitive to judgments. | **Academic motivation**  Objective: This session is dedicated to academic motivation and homework. |
| 14 | **Homework**  Objective: How to best accompany the ADHD child/adolescent during homework. | **Organizing schoolwork and homework**  Objective: This session is dedicated to organizing schoolwork and homework. |
| 15 | **Partnership with the school**  Objective: To inform parents about the possible education arrangements and the different programs made available by the national education system in order to help ADHD children. | **Learning strategies**  Objective: This session is dedicated to memorization strategies and preferred learning modes. |
| 16 | **Screentime**  Objective: To present knowledge concerning the risks and benefits associated with screentime. Emphasize the role parents have in managing screentime. | **Testing strategies**  Objective: How to manage stress associated with academic testing/exams. |
